# Associations of different immune checkpoints-expressing CD4^+^ Treg/ T cell subsets with disease-free survival in colorectal cancer patients

**DOI:** 10.1186/s12885-022-09710-1

**Published:** 2022-06-02

**Authors:** Mohammad A. Al-Mterin, Khaled Murshed, Alhasan Alsalman, Ala Abu-Dayeh, Eyad Elkord

**Affiliations:** 1 Natural and Medical Sciences Research Center, University of Nizwa, P.O. Box 33, Nizwa, 616 Oman; 2 Department of Pathology, Hamad Medical Corporation, Doha, Qatar; 3 Biomedical Research Center, School of Science, Engineering and Environment, University of Salford, Manchester, UK

**Keywords:** FoxP3, Helios, Tregs, Immune checkpoints, DFS, CRC

## Abstract

There are different subsets of T regulatory cells (Tregs), orchestrating critical roles in the regulation of anti-tumor immunity in colorectal cancer (CRC). In this study, we report that a high frequency of circulating CD4^+^FoxP3^+^ Tregs was associated with poorer disease-free survival (DFS), while their higher frequencies in tumor-infiltrating CD4^+^ Tregs was associated with better DFS. We further investigated such associations with four Tregs/T cells expressing or lacking FoxP3 and Helios **(**FoxP3^±^Helios^±^). For the first time, we report that a high frequency of circulating CD4^+^FoxP3^+^Helios^+^ Tregs was associated with poorer DFS, while a high frequency of tumor-infiltrating CD4^+^FoxP3^−^Helios^−^ T cells was associated with poorer DFS. In the four FoxP3^±^Helios^±^ T cell subsets expressing any of the immune checkpoints (ICs) investigated, we found that a high frequency of CD4^+^FoxP3^+^Helios^−^PD-1^+^ Tregs in circulation was associated with worse DFS. We also found that high frequencies of FoxP3^+^Helios^+^CTLA-4^+^ Tregs, FoxP3^+^Helios^−^CTLA-4^+^ Tregs, and FoxP3^−^Helios^+^CTLA-4^+^ CD4^+^ T cells in circulation were associated with worse DFS. In contrast, high frequencies of CD4^+^TIM-3^+^ T cells, FoxP3^+^Helios^+^TIM-3^+^ Tregs, and FoxP3^−^Helios^+^TIM-3^+^ CD4^+^ T cells in circulation were associated with longer DFS. Our data show that certain CD4^+^ Treg/T cell subsets could serve as independent predictive biomarkers in CRC patients. Identification of the exact subpopulations contributing to clinical outcomes is critical for prognoses and therapeutic targeting.

## Introduction

Colorectal cancer (CRC) is the second most deadly malignancy and the third most common cancer in the world [1]. A total of 1.8 million new cases of colon cancer were diagnosed in 2018, accounting for approximately 10% of all new cancer cases and deaths globally [1, 2]. The gastrointestinal tract is susceptible to persistent immune responses, leading to chronic intestinal inflammation, which has a role in the development of cancer, particularly via the secretion of inflammatory cytokines [3]. T regulatory cells (Tregs) are immunosuppressive cells that are found in many different subsets and serve key functions in the maintenance of immunological homeostasis and self-tolerance [4]. They also have critical roles in the regulation of cancer immunity [4]. In different types of cancer, high levels of Treg infiltration into tumors are usually correlated with poor clinical outcomes [5]. However, the role of Tregs is controversial in CRC. Certain studies have shown that tumor-infiltrating FoxP3^+^ Tregs are associated with a better prognosis in CRC patients [6–9]. Conversely, Betts et al., found that high levels of Tregs contribute to disease progression in CRC patients [10]. Helios serves as a marker for T cell activation and proliferation and its expression is required for the maintenance of Treg inhibitory function [11]. In CRC, Helios mRNA level was shown to be higher in tumor tissue in advance stages, suggesting their potential effects in CRC progression [12]. Immune checkpoints (ICs) are critical for immune tolerance and for tissue protection during inflammatory responses [13]. Tumors could modulate the expression of certain ICs as a substantial mechanism of immunological resistance [8, 13].

We have recently reported that some inhibitory ICs, including programmed cell death-1 (PD-1), cytotoxic T lymphocyte-associated antigen (CTLA-4), T cell immunoglobulin and mucin domain-containing protein-3 (TIM-3), and lymphocyte-activation gene-3 (LAG-3), are expressed on CD4^+^ T cells and play roles in CRC progression [8].

This is the first study to insightfully investigate any potential associations between frequencies of different Treg and CD4^+^ T cell subsets expressing ICs with DFS in CRC patients.

## Materials and methods

### Patients and samples

This study was carried out in accordance with ethical approvals (protocol no. MRC-02–18-012) from the Medical Research Center, Hamad Medical Corporation, Doha, Qatar. Peripheral blood samples were collected from thirty-four CRC patients with varying disease stages. Thirty-two patients were eligible and included in the DFS analyses reported in this study. Non-malignant normal tissues (NT) and tumor tissues (TT) from colon were obtained from 22 out of these 32 patients, who underwent surgery at Hamad Medical Corporation, Doha, Qatar. All patients were treatment naive prior to surgery, and they gave written informed consent prior to sample collection.

Clinical and pathological characteristics of these patients are shown in Table 1. All DFS data for the 32 patients were collected in January 2022. Four out of the 32 patients had disease progression, in the form of tumor local recurrence or development of new lymph node and/or distant metastasis. Disease progression was assessed by contrast enhanced chest-abdomen-pelvis computed tomography (CT) scan, that was performed for patients on their clinical follow-up.

**Table1:** Characteristic features of colorectal cancer patients

	**CRC patients**
**Number**	32 [22]^a^
**Median age [range]**	61 [31–96]
**Gender** [Male:Female]	23:9
**TNM stage**	
I	5 [1]^a^
II	9 [8]^a^
III	15 [11]^a^
IV	3 [2]^a^
**MSI-H/dMMR**	4 [3]^a^

*CRC* Colorectal cancer, *MSI-H/dMMR* High MicroSatellite Instability/ deficient MisMatch Repair

^a^Samples used for analyses of tumor-infiltrating lymphocytes

### Multi-parametric flow cytometry

No extra experiments were performed in this study and flow cytometry data of different CD4^+^ T cell subsets were collected. Immune staining and flow cytometry analyses have been done as per our previously published article [8].

### Statistical analyses

Statistical analyses were performed using GraphPad Prism 9 software (GraphPad Software, California, USA). The Shapiro–Wilk test was used to analyze normality of datasets. All immune cell subsets were categorized into low and high groups as below/above mean for normally distributed data, and below/above median for non-normally distributed data. DFS was estimated using the Kaplan–Meier curves, and log-rank test was used to calculate *P* values. *P* value of ≤ 0.05 was considered to be significant.

## Results

### Association of Tregs with DFS

**Fig. 1:**
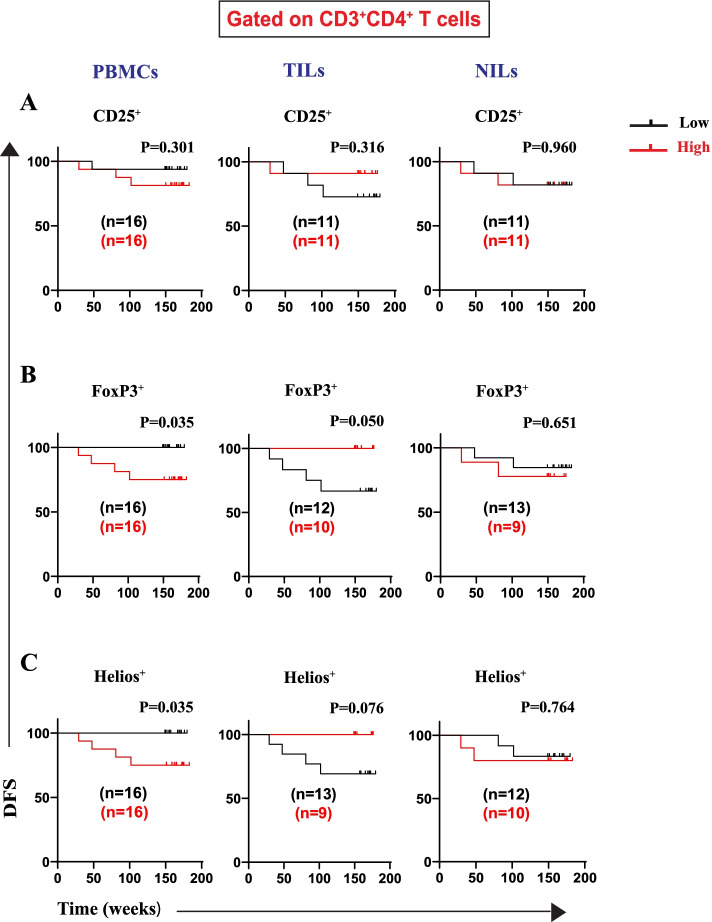
Kaplan–Meier curves of DFS based on frequencies of CD25^+^, FoxP3^+^, and Helios^+^ in PBMCs, TILs, and NILs. Patients with high frequencies of CD25^+^ (**A**), FoxP3^+^ (**B**), and Helios^+^ (**C**), in CD3^+^CD4^+^ T cells, were compared with those with low frequencies of these subsets. Patient numbers investigated are indicated on the survival curves for each cell population in all figures

Tregs are a significant subgroup of CD4^+^ T cells that are characterized by high levels of the interleukin-2 receptor alpha chain (CD25) and the transcription factor forkhead box P3 (FoxP3) [14]. Helios is a transcription factor that modulates FoxP3^+^ Treg functional stability and is needed for their inhibitory action [11, 15–17]. We have previously measured levels of different CD4^+^ T cell subsets in PBMCs, NILs, and TILs of CRC patients [8]. Representative flow cytometric plots and percentages of the different CD4^+^ Treg subsets have already been shown in our previous study [8]. In this study, we further identified the specific Treg subsets contributing to the worse DFS. We found that high frequencies of CD4^+^CD25^+^ T cells were not associated with DFS in circulation or in tissues in this cohort of CRC patients (Fig. 1A). When Tregs were defined based on FoxP3 expression as a more Treg-specific marker, a high Treg frequency was significantly associated with shorter DFS in circulation. Interestingly, a high Treg frequency in TILs, but not in NILs as controls, was associated with longer DFS (Fig. 1B). CD4^+^Helios^+^ T cells showed similar findings like CD4^+^FoxP3^+^ Tregs. More specifically, high frequencies of CD4^+^Helios^+^ T cells showed a significant association with shorter DFS in circulation (Fig. 1C). There were no associations between frequencies of these cell subsets in normal tissues, as controls, with DFS (Fig. 1A-C).

### Association of FoxP3^±^Helios^±^ T cell subsets with DFS

**Fig. 2:**
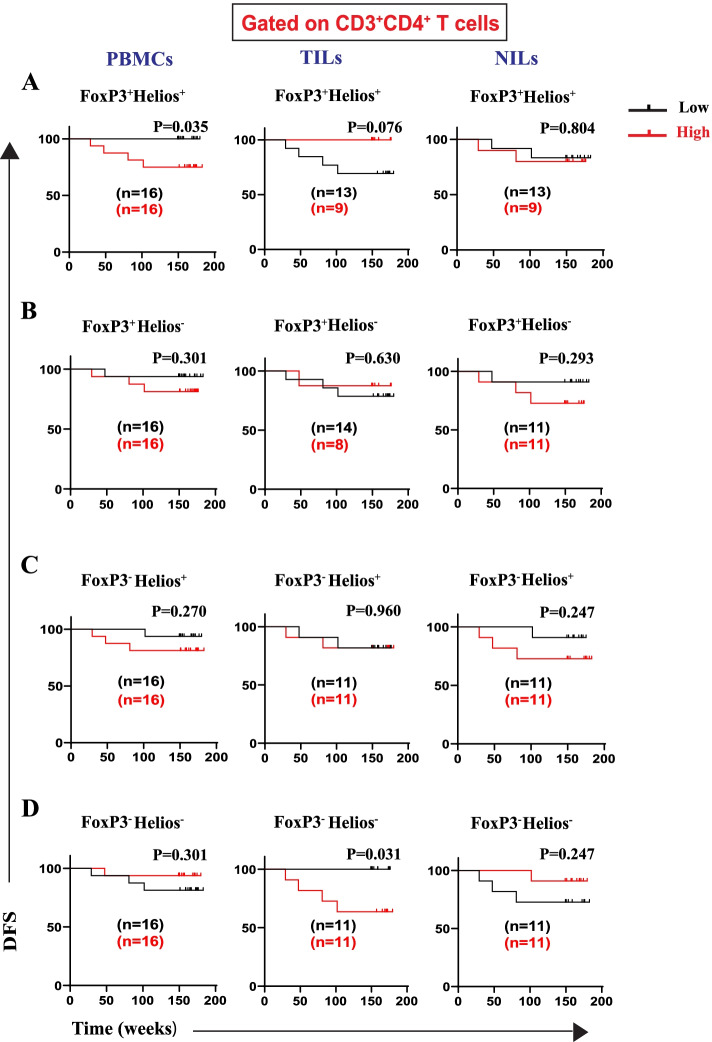
Kaplan–Meier curves of DFS based on frequencies of FoxP3^±^Helios^±^ in PBMCs, TILs, and NILs. Patients with high frequencies of FoxP3^+^Helios^+^ (**A**), FoxP3^+^Helios^−^ (**B**), FoxP3^−^Helios^+^ (**C**), and FoxP3^−^Helios^−^ (**D**), in CD3^+^CD4^+^ T cells, were compared with those with low frequencies of these subsets

We further identified the specific Treg subsets contributing to the worse DFS. We report for the first time, that patients with high frequency of CD4^+^FoxP3^+^Helios^+^ Treg subset in circulation had significantly shorter DFS than patients with low frequency of this Treg subset (Fig. 2A). Additionally, there were no associations between frequencies of both CD4^+^FoxP3^+^Helios^−^ Treg subset and CD4^+^FoxP3^−^Helios^+^ T cells in PBMCs, TILs, and NILs with DFS (Fig. 2B, C). High frequencies of CD4^+^FoxP3^−^Helios^−^ T cells showed a significant association with worse DFS in TILs, while there was no association between frequencies of this T cell subset with DFS in circulation (Fig. 2D). Interestingly, we found that CD4^+^FoxP3^+^Helios^+^ Treg subset and CD4^+^FoxP3^−^Helios^−^ T cell subset in the tumor microenvironment (TME) contributed differently to the DFS in CRC patients (Fig. 2A, D).

### Association of immune checkpoints with DFS

**Fig. 3:**
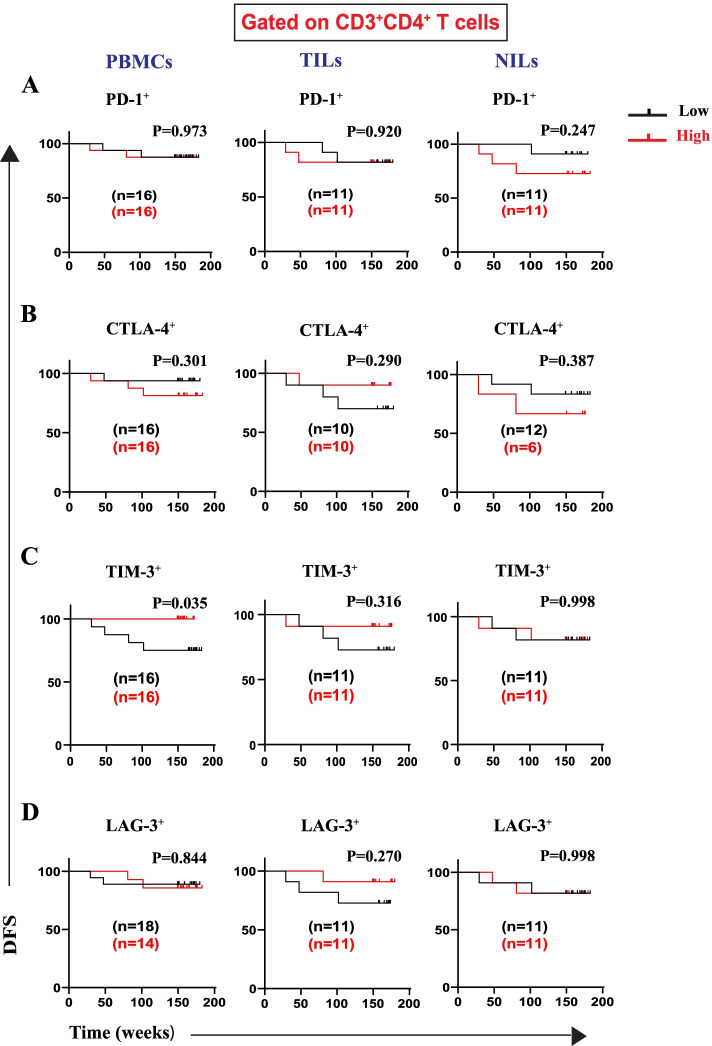
Kaplan–Meier curves of DFS based on frequencies of different immune checkpoints in PBMCs, TILs, and NILs. Patients with high frequencies of PD-1^+^(**A**), CTLA-4^+^ (**B**), TIM-3^+^(**C**), and LAG-3^+^ (**D**), in CD3^+^CD4^+^ T cells, were compared with those with low frequencies of these subsets

During T-cell activation, the immune system uses different checkpoint pathways to maintain co-inhibitory and co-stimulatory signals [8, 18]. Immune checkpoints play critical roles in the inhibition of anti-tumor immune responses in a variety of malignancies, including colorectal cancer [18, 19]. We did not find any association between DFS and frequencies of CD4^+^PD-1^+^ (Fig. 3A) and CD4^+^CTLA-4^+^ T cells (Fig. 3B) in PBMCs, TILs, and NILs. Interestingly, patients with higher frequencies of CD4^+^TIM-3^+^ T cells in circulation showed significantly improved DFS (Fig. 3C). Otherwise, there were no associations between frequencies of CD4^+^TIM-3^+^ T cells with DFS in tumor and normal tissues (Fig. 3C). Additionally, there were no associations between frequencies of CD4^+^LAG-3^+^ T cells in PBMCs and TILs with DFS (Fig. 3D). We did not see any associations between frequencies of these subsets in normal tissues, as controls, with DFS. Overall, CD4^+^TIM-3^+^ T cells seem to be the most critical IC-expressing CD4^+^ T cell subset contributing to DFS in CRC patients.

### Association of different FoxP3^±^Helios^±^ T cell subsets and ICs with DFS

**Fig. 4:**
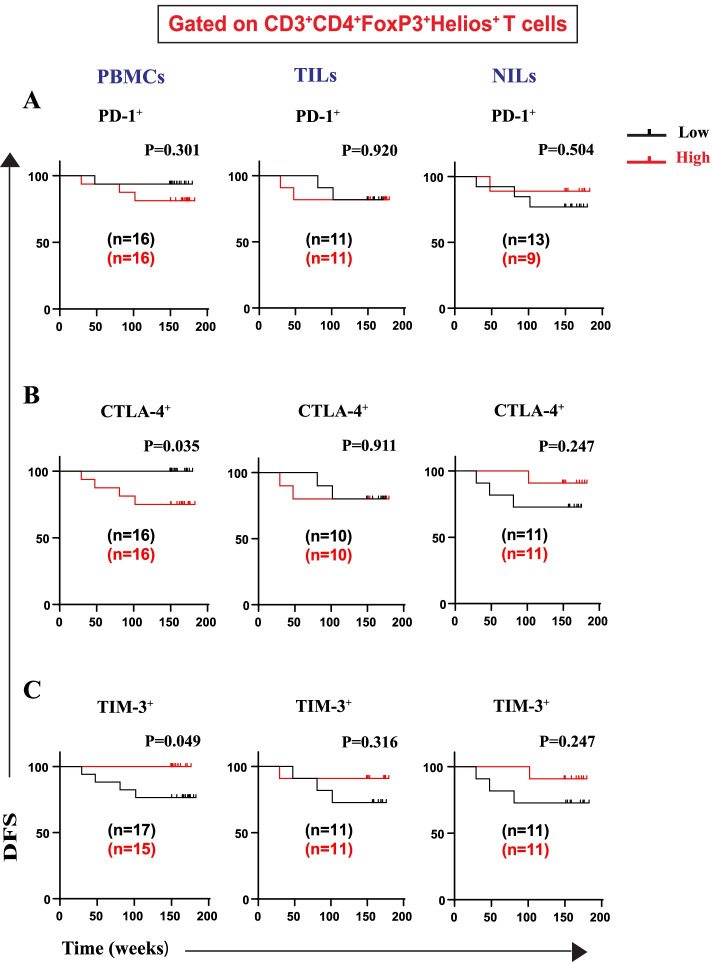
Kaplan–Meier curves of DFS based on frequencies of CD4^+^FoxP3^+^Helios^+^ cells expressing immune checkpoints in PBMCs, TILs, and NILs. Patients with high frequencies of PD-1^+^ (**A**), CTLA-4^+^ (**B**), and TIM-3^+^ (**C**), in CD3^+^CD4^+^FoxP3^+^Helios^+^ T cells, were compared with those with low frequencies of these subsets

**Fig. 5:**
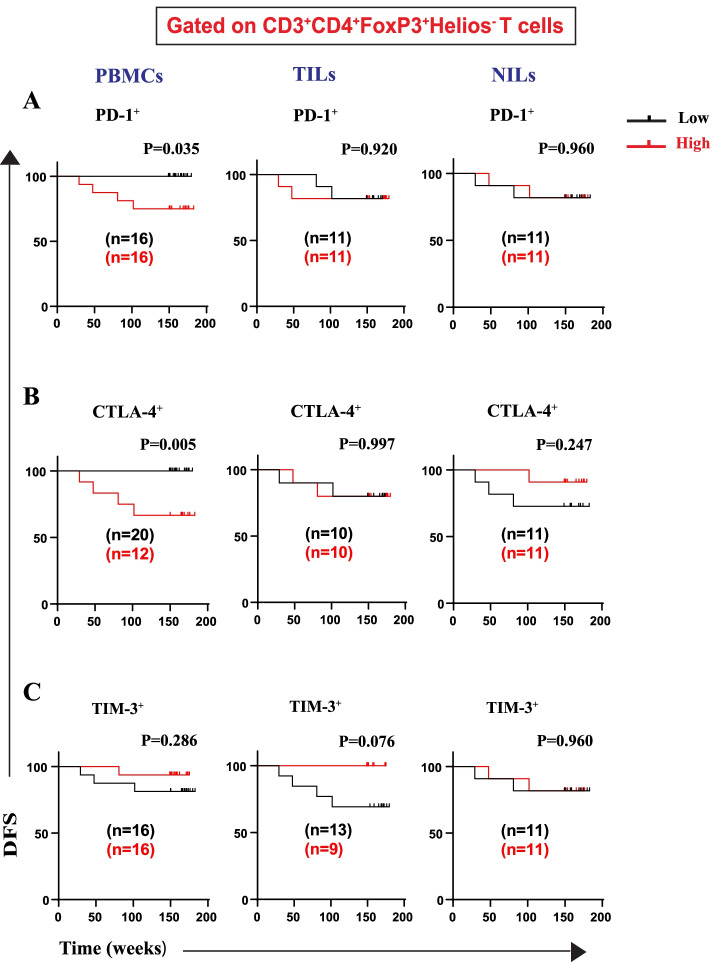
Kaplan–Meier curves of DFS based on frequencies of CD4^+^FoxP3^+^Helios^−^ expressing immune checkpoints in PBMCs, TILs, and NILs. Patients with high frequencies of PD-1^+^ (**A**), CTLA-4^+^ (**B**), and TIM-3^+^ (**C**), in CD3^+^CD4^+^FoxP3^+^Helios^−^ T cells, were compared with those with low frequencies of these subsets

We then went further and identified the specific IC-expressing Treg and T cell subsets, based on the frequency of FoxP3 and Helios (FoxP3^±^Helios^±^), which contribute to worse DFS. We did not find any association between frequencies of CD4^+^FoxP3^+^Helios^+^PD-1^+^ Tregs in PBMCs, TILs, and NILs with DFS (Fig. 4A). We report, for the first time, that CRC patients with higher frequencies of CD4^+^FoxP3^+^Helios^+^CTLA-4^+^ Treg subset in circulation, but not in TILs or NILs, had significantly shorter DFS than patients with lower frequencies of this Treg subset (Fig. 4B). Conversely, high frequencies of CD4^+^FoxP3^+^Helios^+^TIM-3^+^ Treg subset in circulation, but not in TILs or NILs, were associated with better DFS (Fig. 4C). Moreover, we found that CRC patients with high frequencies of CD4^+^FoxP3^+^Helios^−^PD-1^+^ and CD4^+^FoxP3^+^Helios^−^CTLA-4^+^ Treg subsets in PBMCs, but not in TILs or NILs, had significantly shorter DFS than patients with lower frequencies of these Treg subsets (Fig. 5A, B). Moreover, there were no associations between frequencies of CD4^+^FoxP3^+^Helios^−^TIM-3^+^ Treg subset with DFS in PBMCs, NILs and TILs (Fig. 5C).

**Fig. 6:**
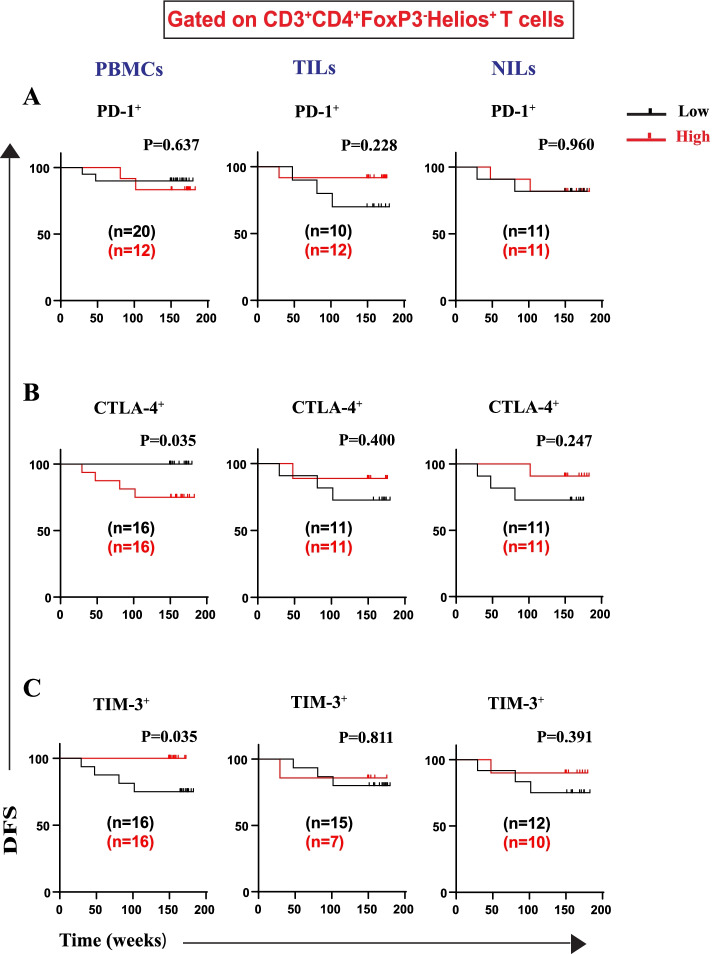
Kaplan–Meier curves of DFS based on frequencies of CD4^+^FoxP3^−^Helios^+^ expressing immune checkpoints in PBMCs, TILs, and NILs. Patients with high frequencies of PD-1^+^ (**A**), CTLA-4^+^ (**B**), and TIM-3^+^ (**C**), in CD3^+^CD4^+^FoxP3^−^Helios^+^ T cells, were compared with those with low frequencies of these subsets

**Fig. 7:**
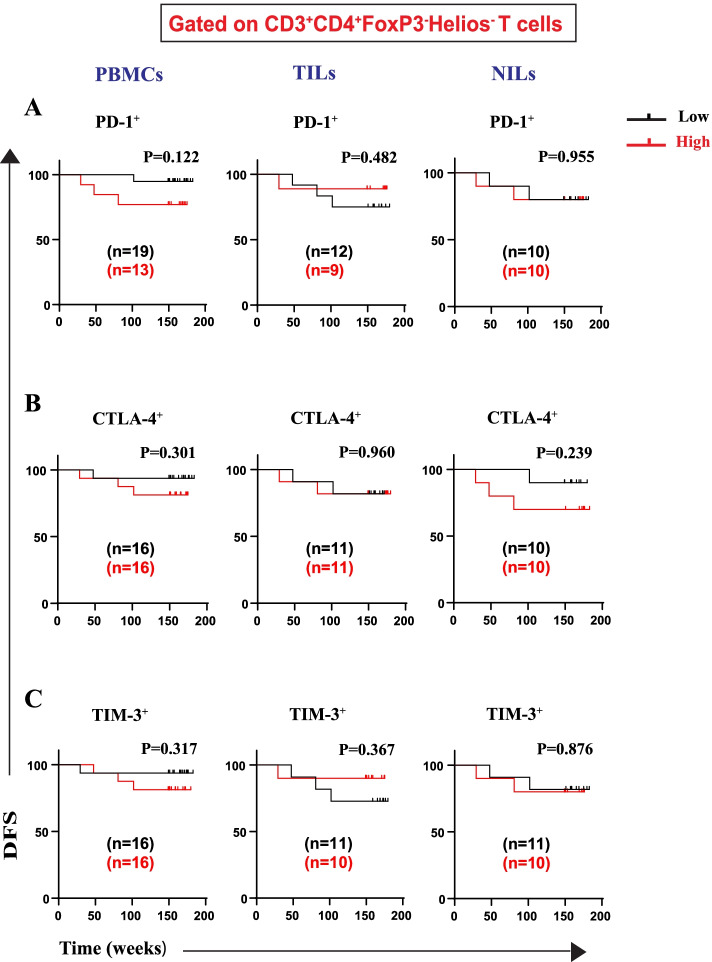
Kaplan–Meier curves of DFS based on frequencies of CD4^+^FoxP3^−^Helios^−^ expressing immune checkpoints in PBMCs, TILs, and NILs. Patients with high frequencies of PD-1^+^ (**A**), CTLA-4^+^ (**B**), and TIM-3^+^ (**C**) in CD3^+^CD4^+^FoxP3^−^Helios^−^ T cells, were compared with those with low frequencies of these cell subsets

Furthermore, we investigated DFS associations with frequencies of IC-expressing CD4^+^FoxP3^−^ T cells (with or without Helios expression) (Figs. 6 and 7). We did not find any association between frequencies of CD4^+^FoxP3^−^Helios^+^PD-1^+^ Tregs in PBMCs, TILs, and NILs with DFS (Fig. 6A). Indeed, patients with high frequencies of CD4^+^FoxP3^−^Helios^+^CTLA-4^+^ T cell in circulation, but not in TILs or NILs, had significantly shorter DFS (Fig. 6B). Moreover, a high frequency of CD4^+^FoxP3^−^Helios^+^TIM-3^+^ T cells in circulation showed a significant association with longer DFS (Fig. 6C). However, there were no associations between frequencies of this T cell subset with DFS in TILs and NILs (Fig. 6C). Of note, there were no associations between frequencies of CD4^+^FoxP3^−^Helios^−^ T cells expressing PD1, or CTLA-4, or TIM-3 with DFS in PBMCs, TILs, and NILs (Fig. 7). Table 2 summarizes associations of high frequencies of any CD4^+^ Treg/T cell subsets with DFS.

**Table 2:** Summary of the significant associations of high frequencies of different CD4^+^ T cell subsets with DFS

CD4^+^ T cell subsets	PBMCs	TILs	NILs
FoxP3^+^	Unfavorable	Favorable	None
Helios^+^	Unfavorable	None	None
FoxP3^+^Helios^+^	Unfavorable	None	None
FoxP3^−^Helios^−^	None	Unfavorable	None
FoxP3^+^Helios^−^PD-1^+^	Unfavorable	None	None
FoxP3^+^Helios^+^CTLA-4^+^	Unfavorable	None	None
FoxP3^+^Helios^−^CTLA-4^+^	Unfavorable	None	None
FoxP3^−^Helios^+^CTLA-4^+^	Unfavorable	None	None
TIM-3^+^	Favorable	None	None
FoxP3^+^Helios^+^TIM-3^+^	Favorable	None	None
FoxP3^−^Helios^+^TIM-3^+^	Favorable	None	None

## Discussion

Immune cells have the ability to recognize and destroy cancer cells. CD4^+^ T cells may target cancer cells by modulating the tumor microenvironment [20, 21]. CD4^+^ T lymphocytes are predominantly T regulatory cells in CRC tissues, and they express several IC molecules such as PD-1, CTLA-4, TIM-3, and LAG-3 [8]. High levels of Treg-related markers were observed in the TME in CRC patients, suggesting their potential effects in carcinogenesis [8, 22, 23]. In contrast to other solid tumors, high levels of tumor-infiltrating FoxP3^+^ Tregs were related with increased survival in CRC patients [9, 24]. Recent studies reported that tumor-infiltrating CD39^+^ Tregs in CRC patients expressed different markers such as OX-40, CTLA-4 and ICOS, implicating their high immunosuppressive abilities in inhibiting anti-tumor immune responses [25, 26]. Correale et al., showed that a higher level of FoxP3^+^ T-lymphocyte tumor infiltration in CRC patients receiving chemotherapy or chemo-immunotherapy was a favorable prognostic marker [27]. Moreover, a high frequency of FoxP3^+^ Tregs within tumor lead to a promising outcome in CRC, suggesting that FoxP3^+^ Tregs are one of the most useful indicators for predicting the prognosis of CRC [7, 28–30]. Another study found that CD8^+^:FoxP3^+^ cell ratios were significantly correlated with distant-recurrence-free survival (DRFS) in the CRC TME [31]. Also, they reported that high numbers of FoxP3^+^ cells were associated with longer overall survival (OS) and DFS, although non-significantly [31]. Interestingly, FoxP3^+^ T cells only have a positive effect on survival in colon tumors that have low levels of CD8^+^ T-cell infiltration [32]. Another study found that TGF-β, which is produced by tumors, has been linked to an increase in the number of intratumoral FoxP3^+^ Tregs [33]. Moreover, they found that intratumoral CD8^+^ T cell:FoxP3^+^ Treg ratio positively correlated with longer DFS and OS [33]. Nevertheless, functionally different subgroups of tumor-infiltrating FoxP3^+^ Tregs contribute in opposing ways to determining CRC disease prognosis [6].

Importantly, it has been demonstrated that circulating Tregs are effective in suppressing antitumor immunity, leading to an adverse outcome of CRC patients [10, 34]. We found that higher frequencies of CD4^+^FoxP3^+^ Tregs in circulation were associated with shorter DFS, implicating the harmful effect of these suppressive cells in inhibiting anti-tumor immune responses in circulation. However, high frequencies of this Treg subset in the TME were associated with longer DFS, indicating the beneficial anti-inflammatory role of CD4^+^FoxP3^+^ Tregs in the TME of CRC patients.

Expression of FoxP3 was highly and positively correlated with the expression of Helios on T cells within PBMCs and TILs in cancer patients [17]. In tumor tissues, the majority of Tregs co-expressed both FoxP3 and Helios, suggesting higher immunosuppressive potentials than cells with single expressions of FoxP3 or Helios [17]. Classification of FoxP3^+^ Tregs into subsets helps to investigate Treg cell differentiation in both normal and disease conditions, as well as to alter immune responses by modulating specific FoxP3^+^ Treg subpopulations [35]. Our group has also recently proposed that FoxP3^+^Helios^+^ Tregs constitute a functional subset of Tregs with higher suppressive characteristics [36]. Tumor tissues in CRC patients were characterized by high levels of Helios^+^ Tregs compared to PBMCs and normal colon tissues [24, 37], suggesting their potential roles in CRC progression [12]. In this study, we found that a high frequency of CD4^+^FoxP3^+^Helios^+^ Tregs in blood was associated with shorter DFS, suggesting the potential role of this highly immunosuppressive Treg subset in inhibiting anti-tumor immune responses, and consequently worsening clinical outcomes. Importantly, it is hypothesized that the TME could enhance the induction of the FoxP3^+^Helios^+^ Treg subset from the FoxP3^−^Helios^+^ T cell subset [17]. Interestingly, we found that high frequencies of CD4^+^FoxP3^−^Helios^−^ TILs were significantly associated with worse DFS, suggesting that these cells could induce inflammation in CRC TME.

In TME, certain tumor ligands bind to inhibitory molecules on T cells, such as CTLA-4, PD-1, TIM-3, and LAG-3 and others, which in turn produce immune-suppressive mediators, leading to the failure of cancer elimination [18, 38]. CD4^+^PD-1^+^ T cells were predominantly detectable in tumor tissues of CRC patients [39], which may lead to T-cell exhaustion and cancer progression [40, 41]. Additionally, CRC patients with high expression of PD-1 had worse TNM staging and DFS, compared with those with low expression [42]. In agreement with these studies, we found that high frequencies of FoxP3^+^Helios^−^PD-1^+^ were associated with shorter DFS in circulation. More samples might be required to determine possible associations of these subsets with DFS in tumor tissues.

TIM-3 is frequently overexpressed on exhausted CD4^+^ T cells in CRC patients, suggesting this could be associated with worse prognoses [43–45]. Moreover, TIM-3 was correlated with CRC progression and might be a possible therapeutic target [46]. Arai et al., found that TIM-3 expression on CD4^+^ T cells was significantly increased after CRC operation [47]. Additionally, they found that the production of IFN-γ was linked to TIM-3 and PD-1 expression on CD4^+^ and CD8^+^ T cells, suggesting that TIM-3^+^PD-1^+^CD4^+^ and CD8^+^ T cells are highly dysfunctional [47]. On the other hand, Zhang et al., found that TIM-3 expression either in the primary or metastatic tumor was associated with better progression-free survival (PFS) in renal cell carcinoma [48]. We found that high frequencies of TIM-3^+^ and FoxP3^+^Helios^+^TIM-3^+^ CD4^+^ T cells were associated with longer DFS in circulation. Of note, most studies investigated TIM-3 expression in bulk tumor tissues but not on specific T cell subsets. This is the first study to indicate that TIM-3 expression on T cells is associated with better DFS in CRC.

We have previously reported that mRNA level of CTLA-4 in tumor tissues was increased in advanced stages of CRC, suggesting their possible effects in CRC progression [12]. Moreover, we have shown that there was a significant elevation in levels of CD4^+^CTLA-4^+^ T cells only in PBMCs of CRC patients with advanced stages, suggesting that there is a relationship between increased levels of CTLA-4^+^ Tregs and CRC progression [8]. In this study, high frequencies of CTLA-4 expressed in different Treg subsets were associated with worse DFS in circulation. Therefore, targeting CTLA-4 on these subsets might have beneficial roles in CRC.

A recent study showed that overexpression of LAG-3 on tumor tissues was associated with worse prognosis in patients with microsatellite instability high (MSI-H) colon cancer [49]. In addition, we have previously reported that mRNA level of LAG-3 was higher in PBMCs of CRC patients than those of healthy controls [12]. Moreover, the frequency of LAG-3 in tumor tissues was associated with differentiation, lymph node metastasis, and invasion in CRC patients [50]. In our study, we found that there were no associations between frequencies of CD4^+^LAG-3^+^ T cells with DFS. Due to weak overall LAG-3 expression in original study [8], more samples are needed to investigate the role of LAG-3 in DFS in CRC patients.

CRC patients with mismatch repair deficiency (dMMR) usually have worse prognosis than patients without dMMR [51]. Notably, over-expressions of FoxP3, IL1β, IL17, TGF-β and IL6 were associated with the microsatellite stability MSS phenotype [52, 53]. It has been shown that a high frequency of tumor-infiltrating FoxP3^+^ Tregs predicts improved survival in mismatch repair-proficient CRC patients [53]. Moreover, in stage II MSS CRC, it has been found that low frequencies of both FoxP3^+^ and CD3^+^ TILs were associated with the highest progression risk [54]. In this study, we were not able to investigate such association because only 4 CRC patients had dMMR (12.5% of this study cohort, as shown in Table 1); the percentage of dMMR patients was low as expected in CRC patients [55].

Our study highlights the potential of some CD4^+^ T cell subsets as predictive biomarkers associated with worse DFS in CRC patients. Overall, high frequency of tumor-infiltrating FoxP3^−^Helios^−^ T cells, and high frequencies of circulating FoxP3^+^, Helios^+^, FoxP3^+^Helios^+^, FoxP3^+^Helios^−^PD-1^+^, FoxP3^+^Helios^+^CTLA-4^+^, FoxP3^+^Helios^−^CTLA-4^+^ Tregs, and FoxP3^−^Helios^+^CTLA-4^+^ T cells, are associated with shorter DFS. Targeting these immune cell subsets in CRC patients could improve clinical outcomes. On the other hand, high frequencies of CD4^+^TIM-3^+^, FoxP3^+^Helios^+^TIM-3^+^ Tregs and FoxP3^−^Helios^+^TIM-3^+^ T cells in circulation are associated with longer DFS in CRC patients, suggesting that T cells expressing TIM-3 could be activated cells with improved anti-tumor activities.

Most available studies investigated expression of ICs in bulk tumor tissues but not on specific CD4^+^ and CD8^+^ T cell subsets. To date, this is the first study to investigate the associations of different CD4^+^ Treg subsets and immune checkpoints-expressing CD4^+^ T cells with DFS in CRC patients. In addition to CD4^+^ T cell subsets in this study, we have also investigated the association of CD8^+^ T cell subsets with DFS in CRC patients (Alsalman et al., submitted for publication). However, multi-center investigations are required to confirm these findings in larger cohorts of patients. Moreover, additional investigations are required to determine the exact function of these cell subsets in the TME and circulation of CRC patients. Our data suggest that different CD4^+^ Treg/T cell subsets in circulation or in the TME play different roles in DFS of CRC patients. Clearly, identification of the exact subpopulations contributing to clinical outcomes is critical for prognosis purposes and therapeutic approaches.

## Data Availability

The datasets used and/or analyzed during the current study are available from the corresponding author on reasonable request.

## Abbreviations

CRC: Colorectal cancer
CTLA-4: Cytotoxic T-lymphocyte-associated antigen 4
DFS: Disease-free survival
FoxP3: Forkhead box P3
ICs: Immune checkpoints
LAG-3: Lymphocyte-activation gene 3
NILs: Normal tissue-infiltrating lymphocytes
NT: Normal tissues
PBMC: Peripheral blood mononuclear cell
PD-1: Programmed cell death-1
TILs: Tumor-infiltrating lymphocytes
TIM-3: T-cell immunoglobulin and mucin domain-3
TME: Tumor microenvironment
Treg: T regulatory cell
TT: Tumor tissues
